# Hybrid *de novo* and haplotype-resolved genome assembly of Vechur cattle — elucidating genetic variation

**DOI:** 10.3389/fgene.2024.1338224

**Published:** 2024-03-06

**Authors:** Poorvishaa V. Muthusamy, Rajesh Vakayil Mani, Shivani Kumari, Manpreet Kaur, Balu Bhaskar, Rajeev Raghavan Pillai, Thankappan Sajeev Kumar, Thapasimuthu Vijayamma Anilkumar, Nongmaithem Sadananda Singh

**Affiliations:** ^1^ School of Biology, Indian Institute of Science Education and Research, Thiruvananthapuram, Kerala, India; ^2^ Kerala Livestock Development Board, Thiruvananthapuram, Kerala, India; ^3^ Division of Experimental Pathology, Sree Chitra Tirunal Institute for Medical Sciences and Technology, Thiruvananthapuram, India

**Keywords:** indicine, genome, trio binning, Vechur, IGF1, HMGA2, CD68, SrGAP1

## Abstract

Cattle contribute to the nutritional needs and economy of a place. The performance and fitness of cattle depend on the response and adaptation to local climatic conditions. Genomic and genetic studies are important for advancing cattle breeding, and availability of relevant reference genomes is essential. In the present study, the genome of a Vechur calf was sequenced on both short-read Illumina and long-read Nanopore sequencing platforms. The hybrid *de novo* assembly approach was deployed to obtain an average contig length of 1.97 Mbp and an N50 of 4.94 Mbp. By using a short-read genome sequence of the corresponding sire and dam, a haplotype-resolved genome was also assembled. In comparison to the taurine reference genome, we found 28,982 autosomal structural variants and 16,926,990 SNVs, with 883,544 SNVs homozygous in the trio samples. Many of these SNPs have been reported to be associated with various QTLs including growth, milk yield, and milk fat content, which are crucial determinants of cattle production. Furthermore, population genotype data analysis indicated that the present sample belongs to an Indian cattle breed forming a unique cluster of *Bos indicus*. Subsequent F_ST_ analysis revealed differentiation of the Vechur cattle genome at multiple loci, especially those regions related to whole body growth and cell division, especially *IGF1*, *HMGA2*, *RRM2*, and *CD68* loci, suggesting a possible role of these genes in its small stature and better disease resistance capabilities in comparison with the local crossbreeds. This provides an opportunity to select and engineer cattle breeds optimized for local conditions.

## Introduction

Cattle contribute to the nutritional needs and economy of a place. The demand for food from animal sources has been rapidly increasing, especially in developing countries. According to OECD-FAO (Organisation for Economic Co-operation and Development and the Food and Agricultural Organization) of the United Nations Agricultural Outlook 2023–2032, global consumption of milk and dairy products is expected to increase by 0.8% per annum to 15.7 kg milk solid equivalents by 2032. To meet this demand, significant improvement in milk yield is required in developing countries, and improvements in feed, health, and genetics will contribute toward that goal in a sustainable manner. Adopting of new technologies or customization of existing technologies is being carried out in many countries. In general, crossbreeding between a highly adapted but with low-productivity indigenous breed and a poorly adapted but highly productive exotic breed and further selection is conducted to develop a high-yielding well-adapted crossbreed. Cross-breeding under better management has shown a manifold increase in milk yield, thereby leading to substantial increase in household income and reduction of greenhouse gas emission. In recent times, the application of genomics has become increasingly helpful and important for implementation of meticulously planned breeding programs for breed improvement exercises, including breed composition assignment. Genomics-based approaches have been successful in developing economical genotyping panels and/or assays for use during genomic selection, including ancestry proportion determination, which is important during breed selection (Meuwissen et al., 2021; Strandén et al., 2022). Genomic information is used in genomic selection, which helps in more accurate prediction of phenotypes at a young age, utilization of information available for distant breeds, and in reduction of cost, time, and number of crosses as compared to traditional breeding methods (Hayes et al., 2013). Recently, climate change has led to increased incidences of higher-intensity heat waves, which leads to another challenge to the cattle breeding efforts as adaptation to heat stress leads to lower efficiency of production and, thus, is unfavorable to the goal of reducing GHG (Strandén et al., 2022). Indicine breeds are known for their resistance to drought, better tolerance to heat and sunlight (Beatty et al., 2006), and disease resistance (Fernandes Júnior et al., 2020). Thus, crossbreeding using indicine breeds with genomic selection approaches offers a high potential to achieve yield improvement goals. However, the lack of genome sequences of indicine cattle becomes a limiting factor in carrying out genomic-based breeding using indicine breeds. The only available previous reference-based genome assemblies of *Bos indicus* cattle (Nellore breed) and other indicine breeds were done using a short-read sequencing platform. For *B. indicus* cattle (Nellore breed), reference-based genome assembly was performed using the SOLiD sequencing platform with very short read lengths of 25 and 50 bases. The recent reference-based genome assembly was carried out using the Illumina platform with read lengths of 150 bases (Canavez et al., 2012; Chakraborty et al., 2023), while there is a high-quality reference genome for taurine cattle breeds (Rosen et al., 2020). Therefore, a quality reference genome of the indicine breed is still lacking.

The biological and economic output efficiency is very important for dairy farmers, and it has been reported that lighter cows provide a comparatively higher economic value based on land (Thompson et al., 2020). It has also been reported that feed efficiency (milk yield per kg feed) was negatively correlated, ranging from −0.18 for wither height to −0.33 for body weight, with body weight and the body measurements ranging from −0.18 for wither height to −0.33 for body weight (Sieber et al., 1988). Thus, an indicine breed with known history in dairy farming and small size would be an important one to study and for crossbreed development. There are around 75 breeds of indicine cattle majorly split between African breeds and Indian breeds. According to the animal genetic resources portal (https://nbagr.icar.gov.in/en/registered-cattle/), there are 53 registered cattle breeds in India. There are phenotypic variations among these breeds. The Vechur breed found in the south-western state of Kerala, where crossbreeding with taurine breeds of cattle has been practiced over the last 6 decades to improve milk production, is a small sized, well-adapted cattle breed with an average weight of about 133.6 ± 3.7 and 173.5 ± 6.8 kg and a height of 89.0 ± 0.7 and 99.8 ± 1.4 cm for cows and bulls, respectively. This was the most popular dairy breed, producing 2–3 L of milk per day in the region before it was replaced by high milk-yielding crossbreeds (Iype, 2013). These cattle are also well-known for their resistance to viral, bacterial, and parasitic diseases compared to the exotic cattle and their crossbreds (Radhika et al., 2018; Shivakumara et al., 2018).

In the present study, we have collected a family trio (sire, dam, and calf) of Vechur cattle. The calf genome was sequenced using both short-read Illumina and long-read nanopore platforms to assemble a genome using a hybrid *de novo* assembly approach. Using short read sequences of the sire and dam, a haplotype-resolved genome was also assembled. Furthermore, genetic variants were analyzed using the taurine breed reference genome to find an association with various QTLs. F_ST_ analysis was carried out using the new genome sequence data and other available genotyping data to find genetic loci that may differentiate Vechur from the rest of the indicine breed and may explain its short stature too.

## Results

### Samples and sequencing

Blood DNA samples of a family trio consisting of a dam (MT435), a sire (MT436), and its calf (MT434) were collected and sequenced on a short-read Illumina sequencing platform. The calf DNA was also sequenced using the Nanopore long-read sequencing platform. The sequencing details are given in Table 1.

**TABLE 1:** Details of sequencing.

Short-read Illumina platform sequencing details						
Sample	Average read length (bp)X2	#Raw reads (forward/reverse)	#Total	Raw data (bp)	%GC	Coverage
Name			Reads			
MT434 (calf)	151	449742276x2	899,484,552	135,822,167,352	44	50.3
MT435 (dam)		432569400x2	865,138,800	130,635,958,800	44	48.4
MT436 (sire)		559344678x2	1,118,689,356	168,922,092,756	44	62.6

Long-read Nanopore platform sequencing details						
Sample Name	Mean read length (bp)	# Total reads (single end)	Raw data (bp)	%GC	Coverage	
MT434	4,273	37,211,638	159,011,500,107	43.73	58.89	

### *De novo* genome assembly

**FIGURE 1:**
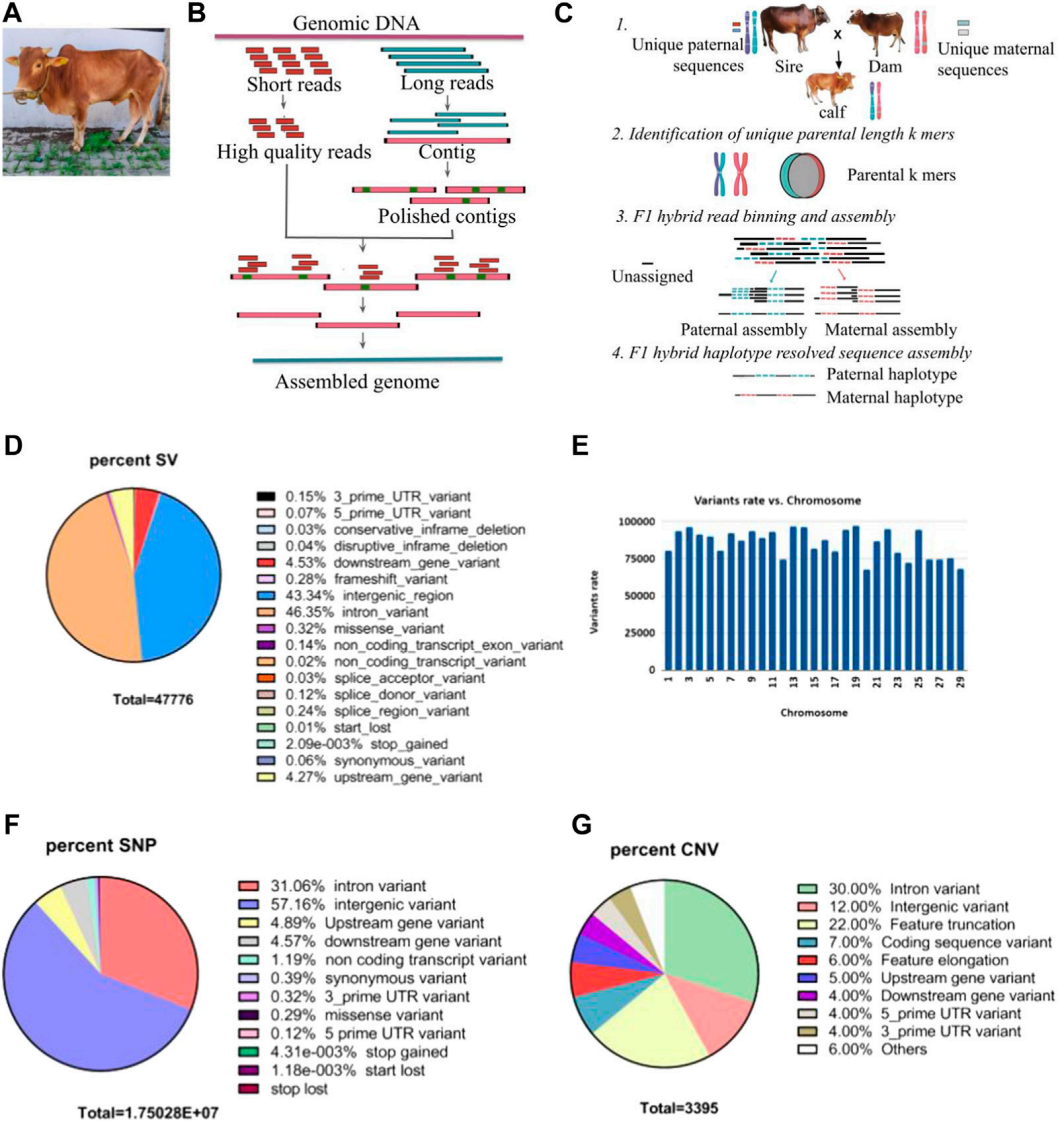
**(A)** A picture of mature Vechur. **(B)** Schematic diagram showing the hybrid de novo genome assembly pipeline. **(C)** Schematic diagram of the trio binning haplotype-resolved genome assembly. **(D)** Pie chart showing the percent of structural variants with each predicted consequences of sample MT434 (calf) obtained using the ensembl variant effect predictor (VEP). **(E)** Histograms showing chromosome-wise structural variant rate. **(F)** Pie chart showing percent of single nucleotide variants (SNVs) with VEP predicted consequences. **(G)** Pie chart showing percent of copy number variants (CNVs) with VEP.

A hybrid *de novo* hybrid assembly was performed for the sample MT434 (calf) using CLC Genomics workbench 22.0.5 using both Nanopore and Illumina reads. For this sample, sequencing by both Illumina and Oxford Nanopore NGS platforms generated raw data of 135 GB and 159 GB, respectively, corresponding to 50.3x and 58.89x coverage, respectively. It was performed in two steps, as depicted in Figure 1B: i) *de novo* assembly of a genome using long, error-prone reads and ii) improve the *de novo* assembly from long reads by polishing with short, high-quality Illumina reads. The refined assembly results in a genome of 2,693,805,279 bp, and the assembly statistics are given in Table 2. To assess the genome completeness further, the Benchmarking Universal Single-Copy Orthologs (BUSCO) (Simão et al., 2015) was used, which has a predefined and expected set of single-copy marker genes as a proxy for genome-wide completeness. The assembled genome was used, and the genome mode was selected, and for lineage, Eukaryote was selected to run just on eukaryote trees to find optimum lineage. The results have been summarized in Table 2.

**TABLE 2:** Assembly statistics and BUSCO analysis summary.

	Sample: MT434
Contigs	1,367
Minimum length	10,281
Maximum length	27,791,687
Average length	1,970,596
N50	4,946,819
N90	1,163,082
Total	2,693,805,279
BUSCO analysis	
BUSCO summary	C: 90.4% [S: 85.1%, D: 5.3%],F: 2.3%, M: 7.3%, n: 303
Complete BUSCOs C	274
Complete and single-copy BUSCOs S	258
Complete and duplicated BUSCOs D	16
Fragmented BUSCOs F	7
Missing BUSCOs M	22
Total BUSCO groups searched	303

### Haplotype-resolved assembly

Haplotype-resolved assemblies were generated using the TrioCanu module of the Canu assembler (Koren et al., 2018). To enable haplotype-resolved assembly of the calf, we performed short-read sequencing of the dam and sire using the Illumina platform with a coverage of 32.58 x and 37.67 x, respectively. These reads were quality-trimmed and filtered. Haplotype binning (trio binning) was conducted which takes the short reads from the parental genomes to partition long reads from the offspring into haplotype-specific sets as depicted in Figure 1C. Details of the binned reads are summarized in Table 3. Using the binned reads, each haplotype was then assembled independently using the Long Read Support (beta) plugin of CLC Genomics workbench 22.0.5. These resulted in a paternal haplotype assembly of 2,556,074,938 bp with an N50 of 1.4 Mbp and a maternal haplotype assembly of 2,618,152,939 bp with an N50 of 2.0 Mbp, as summarized in Table 4.

**TABLE 3:** Summary of haplotype binning (trio binning).

	MT434	
	Reads	Bases
Haplotype 1 (paternal)	11,227,111	68,338,934,720
Haplotype 2 (maternal)	12,768,257	77,999,737,705
No haplotype	3,250,033	7,046,060,552
Ignored (short)	9,966,237	5,626,767,130
Unassigned	Fewer than 5% of bases in unassigned reads; not including them in assemblies	

**TABLE 4:** Summary of haplotype-resolved assemblies.

	MT434_Haplotype1	MT434_Haplotype2
Contigs	4,217	2,956
Minimum	10,055	10,035
Length		
Maximum	10,270,387	10,894,072
Length		
Average	606,136	885,708
Length		
N50	1,373,887	2,074,774
N90	300,353	438,793
Total	2,556,074,938	2,618,152,939

### Structural and single-nucleotide variant analysis

In comparison to the taurine reference genome ARS-UCD 1.2.15, we detected 30,434 structural variants with 28,982 autosomal structural variants ranging from 50 bp to 9.97 kbp, with an average of one structural variant for every 86,363 bp with the highest and lowest mutation rate on chromosome 19 and 20, respectively (Figure 1E). Most of the variants (∼90%) are in the intergenic and intronic regions, while 529 variants (∼1.2%) are in the coding regions (Figure 1D). In addition, there are 16,926,990 single-nucleotide variants with 1,521,747 novel and 15,405,243 existing variants: 5,634,648 (33.3%) in the coding region, 51,390 missense, 754 nonsense variants, and 11 read-through variants (Figure 1F). When analyzed using CNVnator 4.0 (Abyzov et al., 2011), we also detected 3,395 copy number variations (2,470 deletions and 925 duplications), and the VEP analysis predicts 22% feature truncation, 6% feature elongation and 7% coding sequence variant, as seen in Figure 1G. When the PANTHER database (https://www.pantherdb.org/) was used to functionally annotate the 712 genes found in the inferred CNV regions, the most enriched pathways were the IGF pathway-mitogen-activated protein kinase/MAP kinase cascade, T-cell activation, gonadotropin-releasing hormone receptor pathway, and interleukin signaling pathway.

**FIGURE 2:**
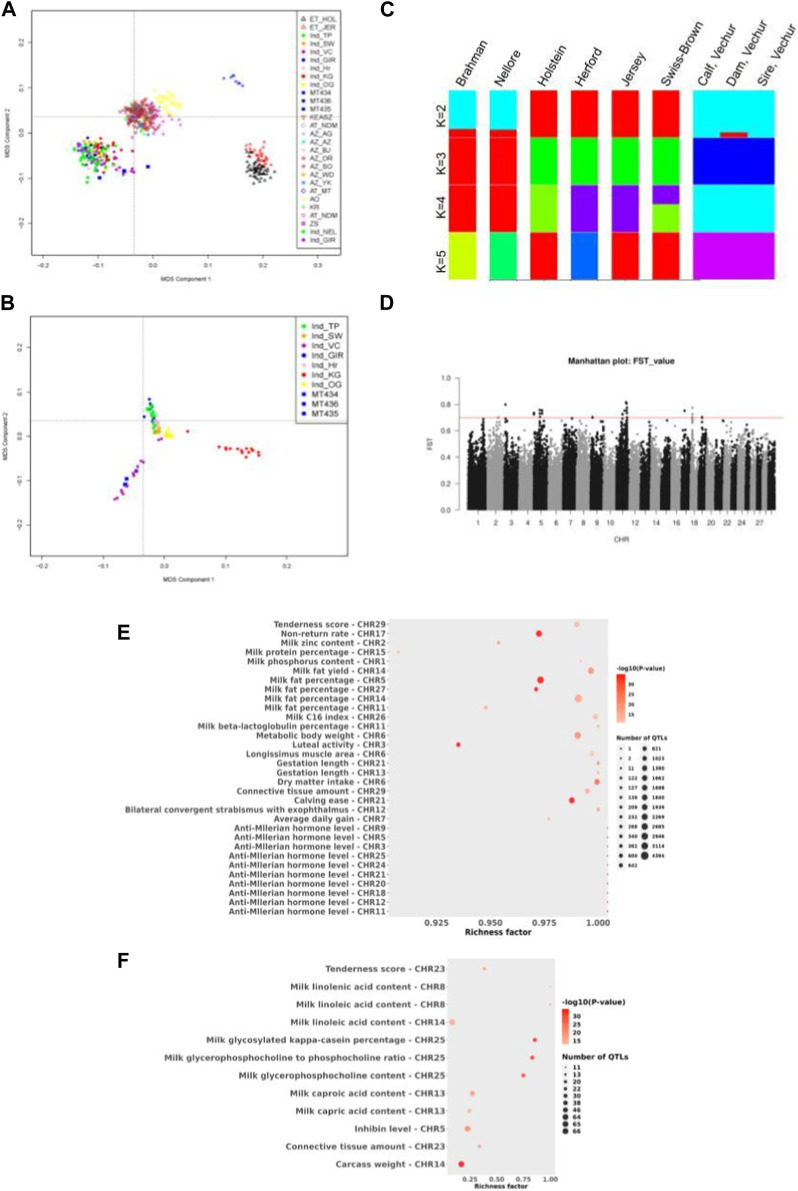
**(A)** Multidimensional scaling (MDS) analysis of various cattle breeds including indicine breeds to show the clustering of Vechur cattle with the indicine breeds. Breed abbreviation as follows: (i) ET_XXX: European taurine breeds, (ii) Ind_xxx: Indian indicine breeds, (iii) MT434/435/MT436 are the trio Vechur being used in this study, (iv) KEASZ: Kenya Small EASZ, (v) AT: African taurine, (vi) AZ_xx: African zebu breeds, (vii) AO: Sanga (viii) KR: Uganda large EASZ, and (ix) ZS: Uganda small EASZ. **(B)** Multidimensional scaling (MDS) analysis of various Indian cattle breeds showing the formation of a separate cluster of Vechur cattle. Breed abbreviation as follows: Ind_TP: Tharparkar, Ind_SW: Sahiwal, Ind_GIR: Gir, Ind_OG: Ongole, Ind_VC: Vechur, Ind_Hr: Hariana, and Ind_KG: Kangayam. **(C)** Admixture analysis of six cattle breeds ranging from K = 2 to K = 5. Breed abbreviation as follows: BRM01, Brahman; NEL01 (Nellore) Indicus; HOL01, Holstein; TAU01, Hereford; JER01, Jersey; MT434 (calf); MT435 (dam); and MT436 (sire) of Vechur breed. **(D)** Manhattan plot of genome-wide F_ST_ values (cut-off value > 0.7) comparing Vechur cattle vs. rest of the Indian breeds. **(E)** Bubble plot depicting QTL enrichment analysis for variations that are homozygous in both the dam and sire. A darker red shade in the circles indicates more significant enrichment, and the area of the circles is proportional to the number of associated QTLs. The *x*-axis represents the richness factor, calculated as the ratio of annotated QTLs to the total number of each QTL in the reference database. **(F)** Bubble plot depicts the enrichment analysis of quantitative trait loci (QTL) for variants identified through FST, showing higher differentiation in Vechur compared to other Indian cattle breeds.

Furthermore, statistical analysis was carried out using the GALLO R package (Fonseca et al., 2020), which gives enriched QTLs of statistical significance. For this, variants at QTL loci in GALLO R were called, and the variants which are homozygous in both the sire and dam were subject to enrichment analysis. As shown in Figure 2E, QTLs related to milk yield, milk quality, metabolic body weight, dry matter intake, etc., were significantly enriched.

### Population analysis

Vechur cattle are well known for its disease resistance, better adaptation for tropical extreme climates, and small stature. Classical multidimensional scaling (MDS) based on pairwise identical-by-state (IBS) distance was performed to understand or validate genetic relatedness and population stratification i) between Vechur and different breeds of cattle worldwide using the data published earlier (Bahbahani et al., 2017) and ii) between Vechur and other Indian indicine breeds using the data published earlier (Dixit et al., 2021) with details of the breeds listed in Table 5. As depicted in Figure 2A, Vechur (Ind_VC) and the present trio (blue squares) cluster together with the other Indian indicine breeds, whereas African zebu (AZs in Figure 2A) breeds and taurine breeds (ETs in Figure 2A) form the other two clusters. Further resolution of the MDS analysis among the Indian indicine breeds, Vechur (ind_VCs, purple circle in Figure 2B) along with the trio (MTxxx, blue squares in Figure 2B), forms a unique subcluster, as depicted in Figure 2B, indicating the presence of a unique selection genetic feature. Admixture analysis also supports the above observation as shown in Figure 2C.

**TABLE 5:** List of different breeds for which genotype or genomic sequence data was used in this study.

Population abbreviation	Population name	Number of samples	Reference
ET_HOL	European taurine-Holstein-Friesian	63	Bahbahani et al., 2017
ET_JER	European taurine-Jersey	36	Bahbahani et al., 2017
Ind_TP	Indian-Tharparkar	17	Dixit et al., 2021
Ind_SW	Indian-Sahiwal	13	Dixit et al., 2021
Ind_VC	Indian-Vechur	16	Dixit et al., 2021
Ind_GIR	Indian-Gir	45	Dixit et al., 2021
Ind_Hr	Indian-Hariana	18	Dixit et al., 2021
Ind_KG	Indian-Kangayam	16	Dixit et al., 2021
Ind_OG	Indian-Ongole	17	Dixit et al., 2021
Ind_NEL	Indian-Nelore	35	Dixit et al., 2021
MT434	Kerala-Vechur	1	This study
MT435	Kerala-Vechur	1	This study
MT436	Kerala-Vechur	1	This study
KEASZ	Small East African shorthorn zebu	92	Bahbahani et al., 2017
AT_NDM	African taurine-N'Dama	24	Bahbahani et al., 2017
AZ_AG	African zebu-Adamawa gudali	25	Bahbahani et al., 2017
AZ_AZ	African zebu-Azawak	2	Bahbahani et al., 2017
AZ_BJ	African zebu-Bunaji	22	Bahbahani et al., 2017
AZ_OR	African zebu-Red bororo	22	Bahbahani et al., 2017
AZ_SO	African zebu-Sokoto gudali	19	Bahbahani et al., 2017
AZ_WD	African zebu-Wadara	3	Bahbahani et al., 2017
AZ_YK	African zebu-Yakanaji	12	Bahbahani et al., 2017
AT_MT	African taurine-Muturu	8	Bahbahani et al., 2017
AO	Sanga-Ankole	25	Bahbahani et al., 2017
KR	Karamojong Zebu	16	Bahbahani et al., 2017
ZS	Serere zebu	13	Bahbahani et al., 2017

Fixation index (F_**ST**_) tests were performed to identify SNPs which are highly differentiated in Vechur as compared to other Indian cattle breeds. This analysis would likely reveal SNPs or genomic regions which are involved in controlling body size. As reported earlier, F_ST_ analysis results show 35 SNPs (listed in Table 6) with high F_ST_ values (>0.7) clustered mainly in certain regions of chromosomes 5, 11, and 18 (Figure 2D), and house protein-coding genes: *IGF1*, *HMGA2*, *SRGAP1*, *APOB*, *ENSBTAG00000020828*, *RRM2*, *ZNF276,* and *CD68* (listed in Table 7).

**TABLE 6:** List of SNPs (in the HDbeadchip) with F_ST_ values > 0.7.

Chr	Pos	Weir_and_Cockerham_F_ST_	Gene	Chr	Pos	Weir_and_Cockerham_F_ST_	Gene
2	8,61,80,784	0.700374		11	8,23,32,335	0.753475	
3	91,32,647	0.798089		11	6,55,70,076	0.751379	
5	4,80,08,400	0.75717		11	8,54,54,517	0.733433	
5	6,64,88,531	0.753664		11	4,93,93,563	0.725848	*ENSBTAG00000020828*
5	6,64,99,710	0.753664		11	4,93,61,395	0.723943	
5	6,65,45,432	0.750791	*IGF1*	11	8,74,66,344	0.700458	
5	5,76,396	0.734675		11	8,74,72,407	0.700458	*RRM2*
5	6,65,47,981	0.729853	*IGF1*	11	8,57,72,933	0.700374	
5	6,65,52,462	0.729853	*IGF1*	11	8,57,82,922	0.700374	
5	6,65,57,413	0.729853	*IGF1*	11	8,57,89,132	0.700374	
5	6,65,62,687	0.729853	*IGF1*	17	2,54,05,683	0.750791	
5	4,80,57,883	0.729551	*HMGA2*	18	1,47,60,377	0.773436	
5	5,40,754	0.723949		18	1,46,47,288	0.725848	*ZNF276*
5	4,99,54,556	0.703359	*SRGAP1*	18	1,46,88,492	0.702313	
9	1,46,80,303	0.702501		18	1,46,92,912	0.702313	
11	7,79,88,513	0.815351	*APOB*	18	1,43,27,200	0.701693	
11	8,23,38,187	0.80554		19	2,79,21,934	0.702313	*CD68*
11	8,56,00,438	0.774					

Gene names are maintained italicised.

**TABLE 7:** Summary of the list of all loci differentiated in the Vechur breed.

Chromosome	Co-ordinates	Gene
5	5:66,532,877–66,604,734	Insulin-like growth factor 1 (*IGF1*)
5	5:48,053,846–48,199,963	High mobility group AT hook2 (*HMGA2*)
5	5:49,812,166–49,969,012	SLIT ROBO Rho GTPase activating protein 1 (*SRGAP1*)
11	11:77,953,380–78,040,118	Apolipoprotein B (*APOB*)
11	11:49,390,250–49,396,081	*ENSBTAG00000020828*
11	11:87,466,581–87,473,817	Ribonucleotide reductase regulatory subunit M2 (*RRM2*)
18	18:14,635,302–14,649,693	Zinc finger protein 276 (*ZNF276*)
19	19:27,921,927–27,923,997	*CD68*

Gene names are maintained italicised.

*IGF1* is involved in growth, and dysfunction of *HMGA2* results in autosomal dominant growth retardation phenotype (Leszinski et al., 2018). *HMGA2* regulates *IGF2* which is a paralog of *IGF1* and known to regulate growth (Abi Habib et al., 2018). Using the sequence data of the present family trio that has been generated during this study, variants were called for these chromosomal locations listed in Table 7. A total of 2,324 variants were detected, with 520 being homozygous in all the members of the family with 27 missense (22 in *APOB* and one each in *HMGA2*, *RRM2*, *IGF1*, and *SRGAP1* and *ENSBTAG00000048587*), five splice site variants (two in *APOB*, one each in *RRM2*, *SRGAP1*, and *HMGA2*), and four 5′UTR and 13 3′UTR (one in *IGF1*, six in *RRM2*, and six in *ENSBTAG00000048587*). We also performed a QTL enrichment analysis on variants with higher F_ST_ values, showing greater differentiation in Vechur compared to other Indian cattle breeds (Dixit et al., 2021), using the GALLO R package (Fonseca et al., 2020). Figure 2F shows that QTLs associated with carcass weight, milk quality, and inhibin levels were highly enriched in Vechur.

## Discussion

A haplotype-resolved genome of an indicine breed has been assembled in this study. There is a significant improvement of the indicine cattle genome as compared to the presently available reference genome, as reported earlier in Canavez et al. (2012) and recently built short-read sequencing-based genome (Chakraborty et al., 2023). The use of relevant reference genomes is important and could have a large impact on studies, especially on detecting signatures of selection, as has been reported earlier (Lloret-Villas et al., 2021). Among 53 cattle breeds of India listed at https://nbagr.icar.gov.in/en/registered-cattle/, Vechur is one of the smallest indicine breeds in the world with exceptional adaptation to the tropical weather conditions. Thus, this genome would help in unraveling genetic factors involved in such adaptation.

MDS plot and admixture analysis revealed that Vechur is one of the indicine breeds and its haplotype-resolved genome would serve as a better reference genome for the local and pan-Indian indicine breed. Most of the dairy cattle breeds in India are crossbreeds between taurine breeds like Jersey and Indian breeds. The availability of an indicine breed reference genome would help in genetic studies related to milk production and local environment adaptation phenotypes using state-of-the-art genomic selection procedures. Moreover, a better understanding of genetic factors may help in applying targeted genome editing technologies to introduce desirable trait-related genetic variants in the genome.

We also found high genetic differentiation in multiple regions of the Vechur breed genome as compared to the other indicine breeds. These regions host genes including *IGF1*, *HMGA2*, *SRGAP1*, *APOB*, *ENSBTAG00000020828*, *RRM2*, *ZNF276*, and *CD68*. *IGF1* is a known growth promoting gene and has been reported to contribute 30%–45% of growth in mice (Liu et al., 1993; Stratikopoulos et al., 2008). *HMGA2-*deficient mice, zebrafish, and horse also show reduced growth (Frischknecht et al., 2015; Lee et al., 2022). The *HMGA2* deficiency phenotype for reduced growth may be explained by its regulation of *IGF2* (Abi Habib et al., 2018), which is again related to *IGF1*. There is one missense *IGF1* variant (T151M) and *HMGA2* (G41C) variant homozygous in all the members of the family. These and other variants in these genes are likely to contribute majorly in the small stature phenotype of this cattle breed. *CD68* is a macrophage marker and is reported to be involved in inflammatory reactions (Holness and Simmons, 1993). We believe this Vechur genome assembly will provide genomic resources for evolutionary studies in combination with the other bovine species. Overall, a haplotype-resolved genome of an Indian indicine cattle is reported in this study and will help in genomic selection studies related to improved milk yield, improved efficiency, and better adaptation.

## Materials and methods

### DNA isolation from blood samples

Two millilitres of blood samples were taken in a 15-mL Falcon tube, and 4 mL of chilled lysis buffer (150 mM NH_4_Cl, 10 mM 1M KHCO_3_, and 0.1 mM EDTA) was added. It was kept on ice for 10 min after mixing. It was then centrifuged at 7,000 rpm for 10 min at 4°C. The supernatant was discarded, and the process was repeated until the pellet is clear of RBC (washing two to three times is sufficient). A total of 300 μL of extraction buffer (400 mM NaCl, 2 mM EDTA, 10 mM TrisCl pH 8.0) was added and mixed well. A total of 100 µL of proteinase K (0.2 mg/mL) and 125 µL of 20% SDS was added, mixed, and incubated at 56°C for 6 hours or overnight. Phenol chloroform extraction was performed by adding 500 µL of phenol–chloroform–isoamylalcohol (25:24:1) to the mixture and mixed well by gently inverting the tube up and down for 10 min to get a milky emulsion. Then, the mixture was centrifuged at 10,000 rpm for 6 mins, and the upper aqueous phase was gently extracted again with 500 µL of chloroform–isoamylalcohol (24:1). The DNA was precipitated by adding 1/10th volume of 3M sodium acetate (of an aqueous layer) and 2.5 times volume of chilled absolute alcohol followed by centrifugation first at 10,000 rpm for 5 min and then at 12,000 rpm for next 5 min and finally at 14,000 rpm for 10 min at 4°C. The pelleted DNA was washed two times with 300 µL of 70% ice cold ethanol and dried at room temperature. It was then dissolved in 100 µL nuclease-free water or 1x TE buffer by incubating at 56°C for 10 min. The DNA was then stored at −20°C until further use.

### Sequencing

Extracted DNA was sequenced on both the Illumina and Oxford Nanopore platforms. The short reads produced by Illumina technology were used to estimate genome size and correct errors in the assembled genome. Long reads from the Oxford Nanopore device, on the other hand, were used in the actual genome assembly process. For the Illumina platform, the library was prepared using the Illumina DNA Prep kit 20060060 and sequenced on the Illumina Novaseq 6000 sequencer using S4 flowcell and Novaseq 6000 S4 reagent kit v1.5 (300 cycles). In addition, another library with an average length of 20 kilobases was created using the Oxford Nanopore platform in line with the manufacturer’s instructions. The library was prepared using the Nanopore Ligation sequencing kit and sequenced on the PromethION 24 (P24) platform using FLO-PRO002 R9.4.1 as well as FLO-PRO112 R10.4.

### Genome assembly

The “*De Novo* Assemble Long Reads” tool within CLC Genomics Workbench version 22.0.5 was used with a specialized plugin for *de novo* hybrid assembly. This tool is designed for processing long, error-prone reads, like those from Oxford Nanopore Technologies. It uses open-source components: minimap2, miniasm, raven, and racon. The hybrid assembly involves two main steps: first, the *de novo* assembly of a genome using long, error-prone reads and second, the refining of the initial *de novo* assembly produced from long reads using short, high-fidelity reads.

The uncorrected nanopore reads were used directly. The process begins with finding overlap alignments among the input reads using miniasm/minimap2. These overlaps are preprocessed with pile-o-grams, creating an assembly graph, which is then simplified to produce contigs using the raven assembler. The default settings (k = 15, w = 5, minimum contig size = 1000) and two rounds of racon polishing were applied. Contig polishing is performed twice using racon/minimap2, which improves a partial order alignment (POA = 500) of the reads against the contigs and contig quality through rapid consensus calling.

The assembly was further polished with high-quality Illumina short reads using racon and enhancements from minipolish. Racon uses a divide-and-conquer strategy for rapid consensus calling. Trimmomatic 0.39 was used to trim and filter Illumina reads for quality and length. These reads were then mapped to assembled contigs to refine them. Most contigs had roughly 40 x coverage or higher. The binned reads for individual contigs were retrieved and used for polishing. The partial order alignment (POA) window was set to 500 bp, and the minimum sequence length for output was 10,000 bp, as all contigs were longer. The remaining settings remained consistent.

### Haplotype-resolved assembly

Haplotype-resolved assemblies were also prepared using the TrioCanu module of the Canu assembler (Koren et al., 2018). Prior to assembly, haplotype binning (trio binning) was conducted, which takes the short reads from the parental genomes to partition long reads from the offspring into haplotype-specific sets. Each haplotype is then assembled independently, resulting in a complete diploid reconstruction. For MT434, the parental reads MT435-dam and MT436-sire were quality-trimmed and filtered and then are used for trio binning using the long reads of their offspring MT434. The trio binning divides the total reads into paternal and maternal groups on the basis of the presence of the haplotype-specific k-mers in those bins. These haplotypes were then assembled using the Long Read Support (beta) plugin of CLC Genomics workbench 22.0.5.

### Structural variant analysis

The initial draft assembly was aligned using NUCmer (l = 100, c = 500) against the reference genome *Bos taurus* (cattle)–Hereford breed (ARS-UCD 1.2; GCF_002263795.1) to obtain a delta file, which was then uploaded to Assemblytics to analyze alignments. The input file (OUT.delta.gz) has been provided for loading on the Assemblytics web server and can be used to view the results dynamically. An Ensembl Variant Effect Predictor (McLaren et al., 2016) was used to predict the consequences of the structural variants.

### Alignments and variant identification

Prior to mapping, adapter sequences and low-quality reads were removed using Trimmomatic 0.39, and high-quality reads were aligned to the UMD3.1 bovine reference genome assembly using the BWA-MEM option of Burrows–Wheeler Alignment program (BWA) version 0.7.5a with default parameters (Li, 2013). Following alignment, SAMtools (version 1.9) (Danecek et al., 2021) was used to convert the SAM files to binary format (BAM, Binary Alignment Map) sorting of the mapped reads according to chromosome position. Duplicate reads were filtered from the sorted BAM files using the Picard tool’s MarkDuplicates program (v2.17.11). The single-nucleotide polymorphisms (SNPs) were discovered using the HaplotypeCaller function of the Genome Analysis Toolkit (GATK, version 3.8). All SNPs were filtered using GATK’s “VariantFiltration” with preliminary filter settings of “QUAL <30.0, QualByDepth (QD) < 2.0, Fisher’s exact test (FS) > 60.0, RMS Mapping Quality (MQ) < 40.0, StrandOddsRatio (SOR) > 3.0, MappingQualityRankSumTest (MQRankSum) < −12.5, and ReadPosRankSumTest (ReadPosRankSum) < −8.0>”.

### QTL enrichment analysis

To better understand the unique traits of Vechur cattle, we developed an in-house script to identify genetic variations based on homozygosity in both the dam (MT436) and sire (MT435). Additionally, we used the ‘GALLO’ package in R (Fonseca et al., 2020) for QTL enrichment analysis of homozygous altered allele SNPs in both the dam and sire. QTL annotations for these SNPs were obtained using the ‘find_genes_qtls_around_markers’ function with a GFF file from The Animal QTL Database aligned to the ARS_UCD1.2 reference genome. QTL boundaries were set within 100 kB upstream and downstream of each significant SNP. The enrichment analysis involved computing adjusted *p*-values (Padj values) through a false discovery rate (FDR) with a chromosome-based technique. Traits associated with specific chromosomes and with a Padj value below 0.05 were considered. Visualization of chromosome-enriched traits with significant Padj values was facilitated using the “QTLenrich_plot” function.

### Copy number variation (CNV) detection

The read depth-based CNVnator approach (Abyzov et al., 2011) was employed to determine genomic CNVs between the Vechur sample (MT434) and the ARS-UCD 1.2 bovine reference assembly. According to the author’s recommendations, CNVnator was run on sorted BAM files with a bin size of 100 bp. Following calling, raw CNVs were subjected to quality control to retain confident CNVs. The filtering criteria were *p*-value <0.001 (calculated using *t*-test statistics) and q0 (fraction of mapped reads with zero quality) < 0.5. The genes found in the inferred CNV regions were retrieved and functionally annotated using PANTHER (https://www.pantherdb.org/) (Nikolsky and Bryant, 2009).

### Population structure analysis

To validate genetic relatedness and population stratification, along with our samples, previously reported data comprising 112 individuals of various *B. indicus* breeds were used as the reference (Dixit et al., 2021). These reference populations include Sahiwal (13), Tharparkar (17), Gir (15), Ongole (17), Hariana (18), Kangayam (16), and Vechur (16). Both the datasets were merged using the “vcf merge” tools of VCFtools (Danecek et al., 2011), and only common SNPs in both datasets were preserved. Then, using the program PLINK (version 1.07) (Purcell et al., 2007), we performed classical MDS based on pairwise IBS distance and rendered the plot using the R package MDS plot.

Linkage pruning was also performed for Admixture analysis using PLINK (Purcell et al., 2007), with parameter: indeppairwise = 50 10 0.1, which performs linkage pruning with a window size of 50 kb, window step size of 10 bp, and r2 threshold of 0.1 (i.e., the linkage acceptable threshold). This stage chose a group of independent variants to reduce redundancy. Admixture v1.3.0 (Alexander et al., 2009) was then used to read the PLINK bed file with the default parameters (cross-validation, cv = 5) and cluster number *k*) ranging from 2 to 5. The findings are plotted using R script.

### Screening of differentially selected regions

We employed F_ST_ to detect positive selection signatures in the Vechur genome based on whole-genome SNPs, and other individuals of various *B. indicus* breeds were used as the reference from previously published data (Dixit et al., 2021). First, the mean F_ST_ value according to Weir and Cockerham’s pairwise estimator approach (Weir and Cockerham, 1984) was determined in autosomal chromosomes using VCFtools (v.0.1.13) (Danecek et al., 2011) with default parameters. Genes in the genomic regions with high Z-transformed F_ST_ value (>7.5) were used to identify their functions in terms of Gene Ontology. The results of population differentiation were visualized in the form of a Manhattan plot by the qqman R package (Turner, 2018).

## Data Availability

The datasets presented in this study can be found in online repositories. The names of the repository/repositories and accession number(s) can be found at: https://www.ncbi.nlm.nih.gov/, PRJNA957582.

## Abbreviations

BUSCO, Benchmarking Universal Single-Copy Ortholog; BWA, Burrows–Wheeler alignment program; F_**ST,**_ fixation index; GHG, greenhouse gases; IBS, identical-by-state; MDS, classical multidimensional scaling; NGS, next-generation sequencing; OECD-FAO, Organization for Economic Co-operation and Development and the Food and Agricultural Organization; POA, partial order alignment; QTLs, quantitative trait locus; SNPs, single-nucleotide polymorphisms; SNVs, single-nucleotide variations.

## References

[B1] Abi Habib W. Brioude F. Edouard T. Bennett J. T. Lienhardt-Roussie A. Tixier F. (2018). Genetic disruption of the oncogenic HMGA2–PLAG1–IGF2 pathway causes fetal growth restriction. Genet. Med. 20, 250–258. 10.1038/gim.2017.105 28796236 PMC5846811

[B2] Abyzov A. Urban A. E. Snyder M. Gerstein M. (2011). CNVnator: an approach to discover, genotype, and characterize typical and atypical CNVs from family and population genome sequencing. Genome Res. 21, 974–984. 10.1101/gr.114876.110 21324876 PMC3106330

[B3] Alexander D. H. Novembre J. Lange K. (2009). Fast model-based estimation of ancestry in unrelated individuals. Genome Res. 19, 1655–1664. 10.1101/gr.094052.109 19648217 PMC2752134

[B4] Bahbahani H. Tijjani A. Mukasa C. Wragg D. Almathen F. Nash O. (2017). Signatures of selection for environmental adaptation and zebu × taurine hybrid fitness in east african shorthorn zebu. Front. Genet. 8, 68. 10.3389/fgene.2017.00068 28642786 PMC5462927

[B5] Beatty D. T. Barnes A. Taylor E. Pethick D. McCarthy M. Maloney S. K. (2006). Physiological responses of *Bos taurus* and *Bos indicus* cattle to prolonged, continuous heat and humidity. J. Anim. Sci. 84, 972–985. 10.2527/2006.844972x 16543576

[B6] Canavez F. C. Luche D. D. Stothard P. Leite K. R. M. Sousa-Canavez J. M. Plastow G. (2012). Genome sequence and assembly of *Bos indicus* . J. Hered. 103, 342–348. 10.1093/jhered/esr153 22315242

[B7] Chakraborty A. Bisht M. S. Saxena R. Mahajan S. Pulikkan J. Sharma V. K. (2023). Genome sequencing and *de novo* and reference-based genome assemblies of *Bos indicus* breeds. Genes Genomics 45, 1399–1408. 10.1007/s13258-023-01401-w 37231295

[B8] Danecek P. Auton A. Abecasis G. Albers C. A. Banks E. DePristo M. A. (2011). The variant call format and VCFtools. Bioinformatics 27, 2156–2158. 10.1093/bioinformatics/btr330 21653522 PMC3137218

[B9] Danecek P. Bonfield J. K. Liddle J. Marshall J. Ohan V. Pollard M. O. (2021). Twelve years of SAMtools and BCFtools. GigaScience 10, giab008. 10.1093/gigascience/giab008 33590861 PMC7931819

[B10] Dixit S. P. Bhatia A. K. Ganguly I. Singh S. Dash S. Sharma A. (2021). Genome analyses revealed genetic admixture and selection signatures in *Bos indicus* . Sci. Rep. 11, 21924. 10.1038/s41598-021-01144-2 34753978 PMC8578574

[B11] Fernandes Júnior G. A. de Oliveira H. N. Carvalheiro R. Cardoso D. F. Fonseca L. F. S. Ventura R. V. (2020). Whole-genome sequencing provides new insights into genetic mechanisms of tropical adaptation in Nellore (Bos primigenius indicus). Sci. Rep. 10, 9412. 10.1038/s41598-020-66272-7 32523018 PMC7287098

[B12] Fonseca P. A. S. Suárez-Vega A. Marras G. Cánovas Á. (2020). GALLO: an R package for genomic annotation and integration of multiple data sources in livestock for positional candidate loci. GigaScience 9. 10.1093/gigascience/giaa149 PMC7772745 33377911

[B13] Frischknecht M. Jagannathan V. Plattet P. Neuditschko M. Signer-Hasler H. Bachmann I. (2015). A non-synonymous HMGA2 variant decreases height in shetland ponies and other small horses. PLOS ONE 10, e0140749. 10.1371/journal.pone.0140749 26474182 PMC4608717

[B14] Hayes B. J. Lewin H. A. Goddard M. E. (2013). The future of livestock breeding: genomic selection for efficiency, reduced emissions intensity, and adaptation. Trends Genet. 29, 206–214. 10.1016/j.tig.2012.119 23261029

[B15] Holness C. L. Simmons D. L. (1993). Molecular cloning of CD68, a human macrophage marker related to lysosomal glycoproteins. Blood 81, 1607–1613. 10.1182/blood.v81.6.1607.bloodjournal8161607 7680921

[B16] Iype S. (2013). Vechur cattle – from extinction to sustainability. Anim. Genet. Resour. Génétiques Anim. Genéticos Anim. 52, 105–110. 10.1017/S2078633612000501

[B17] Koren S. Rhie A. Walenz B. P. Dilthey A. T. Bickhart D. M. Kingan S. B. (2018). *De novo* assembly of haplotype-resolved genomes with trio binning. Nat. Biotechnol. 36, 1174–1182. 10.1038/nbt.4277 PMC6476705 30346939

[B18] Lee M. O. Li J. Davis B. W. Upadhyay S. Al Muhisen H. M. Suva L. J. (2022). Hmga2 deficiency is associated with allometric growth retardation, infertility, and behavioral abnormalities in mice. G3 GenesGenomesGenetics 12, jkab417. 10.1093/g3journal/jkab417 PMC9210324 34878116

[B19] Leszinski G. S. Warncke K. Hoefele J. Wagner M. (2018). A case report and review of the literature indicate that HMGA2 should be added as a disease gene for Silver-Russell syndrome. Gene 663, 110–114. 10.1016/j.gene.2018.04.027 29655892

[B20] Li H. (2013). Aligning sequence reads, clone sequences and assembly contigs with BWA-MEM. arXiv. 10.48550/ARXIV.1303.3997

[B21] Liu J. P. Baker J. Perkins A. S. Robertson E. J. Efstratiadis A. (1993). Mice carrying null mutations of the genes encoding insulin-like growth factor I (Igf-1) and type 1 IGF receptor (Igf1r). Cell 75, 59–72. 10.1016/s0092-8674(05)80084-4 8402901

[B22] Lloret-Villas A. Bhati M. Kadri N. K. Fries R. Pausch H. (2021). Investigating the impact of reference assembly choice on genomic analyses in a cattle breed. BMC Genomics 22 (1), 363. 10.1186/s12864-021-07554-w 34011274 PMC8132449

[B23] McLaren W. Gil L. Hunt S. E. Riat H. S. Ritchie G. R. S. Thormann A. (2016). The ensembl variant effect predictor. Genome Biol. 17, 122. 10.1186/s13059-016-0974-4 27268795 PMC4893825

[B24] Meuwissen T. van den Berg I. Goddard M. (2021). On the use of whole-genome sequence data for across-breed genomic prediction and fine-scale mapping of QTL. Genet. Sel. Evol. 53, 19. 10.1186/s12711-021-00607-4 33637049 PMC7908738

[B25] Nikolsky Y. Bryant J. (2009). Protein networks and pathway analysis. Preface. Methods Mol. Biol. 563, v–vii. 10.1007/978-1-60761-175-2 19760825

[B26] Purcell S. Neale B. Todd-Brown K. Thomas L. Ferreira M. A. R. Bender D. (2007). PLINK: a tool set for whole-genome association and population-based linkage analyses. Am. J. Hum. Genet. 81, 559–575. 10.1086/519795 17701901 PMC1950838

[B27] Radhika G. Aravindakshan T. V. Jinty S. Ramya K. (2018). Evaluation of genetic diversity, population structure, and relationship between legendary vechur cattle and crossbred cattle of Kerala state, India. Anim. Biotechnol. 29, 50–58. 10.1080/10495398.2017.1297719 28358589

[B28] Rosen B. D. Bickhart D. M. Schnabel R. D. Koren S. Elsik C. G. Tseng E. (2020). *De novo* assembly of the cattle reference genome with single-molecule sequencing. GigaScience 9, giaa021. 10.1093/gigascience/giaa021 32191811 PMC7081964

[B29] Shivakumara P. N. Aravindakshan T. V. Naicy T. Anilkumar K. Uma R. (2018). Molecular characterization and differential mRNA expression profiling of Toll-like receptor-2 gene in Vechur (*Bos indicus*) and crossbred (*Bos indicus* X *Bos taurus*) cattle of Kerala in response to anthrax vaccination. Meta Gene 16, 15–20. 10.1016/j.mgene.2018.01.003

[B30] Sieber M. Freeman A. E. Kelley D. H. (1988). Relationships between body measurements, body weight, and productivity in Holstein dairy cows. J. Dairy Sci. 71, 3437–3445. 10.3168/jds.s0022-0302(88)79949-x

[B31] Simão F. A. Waterhouse R. M. Ioannidis P. Kriventseva E. V. Zdobnov E. M. (2015). BUSCO: assessing genome assembly and annotation completeness with single-copy orthologs. Bioinformatics 31, 3210–3212. 10.1093/bioinformatics/btv351 26059717

[B32] Strandén I. Kantanen J. Lidauer M. H. Mehtiö T. Negussie E. (2022). Animal board invited review: genomic-based improvement of cattle in response to climate change. Animal 16, 100673. 10.1016/j.animal.2022.100673 36402112

[B33] Stratikopoulos E. Szabolcs M. Dragatsis I. Klinakis A. Efstratiadis A. (2008). The hormonal action of IGF1 in postnatal mouse growth. Proc. Natl. Acad. Sci. 105 (49), 19378–19383. 10.1073/pnas.0809223105 19033454 PMC2614769

[B34] Thompson L. R. Beck M. R. Buskirk D. D. Rowntree J. E. McKendree M. G. S. (2020). Cow efficiency: modeling the biological and economic output of a Michigan beef herd. Transl. Anim. Sci. 4, txaa166. 10.1093/tas/txaa166 33381709 PMC7751152

[B35] Turner S. D. (2018). qqman: an R package for visualizing GWAS results using Q-Q and manhattan plots. J. Open Source Softw. 3, 731. 10.21105/joss.00731

[B36] Weir B. S. Cockerham C. C. (1984). ESTIMATING F -STATISTICS FOR THE ANALYSIS OF POPULATION STRUCTURE. Evolution 38, 1358–1370. 10.1111/j.1558-5646.1984.tb05657.x 28563791

